# Cannabinoid accumulation in hemp depends on ROS generation and interlinked with morpho-physiological acclimation and plasticity under indoor LED environment

**DOI:** 10.3389/fpls.2022.984410

**Published:** 2022-10-05

**Authors:** Md Jahirul Islam, Byeong Ryeol Ryu, Md Hafizur Rahman, Md Soyel Rana, Eun Ju Cheong, Myeong-Hyeon Wang, Jung-Dae Lim, Mohammad Anwar Hossain, Young-Seok Lim

**Affiliations:** ^1^ Department of Bio-Health Convergence, College of Biomedical Science, Kangwon National University, Chuncheon, South Korea; ^2^ Physiology and Sugar Chemistry Division, Bangladesh Sugarcrop Research Institute, Pabna, Bangladesh; ^3^ Division of Forest Science, College of Forest and Environmental Sciences, Kangwon National University, Chuncheon, South Korea; ^4^ Department of Herbal Medicine Resource, Kangwon National University, Samcheok, South Korea; ^5^ Department of Genetics and Plant Breeding, Bangladesh Agricultural University, Mymensingh, Bangladesh

**Keywords:** cannabis, reactive oxygen species, cellular stress, cannabinoid accumulation, LED light composition

## Abstract

Manipulation of growth and development of cannabis (*Cannabis sativa* L.) has received considerable interest by the scientific community due to its high value in medicinal and recreational use worldwide. This study was conducted to investigate the effects of LED spectral changes on reactive oxygen species (ROS) and cannabinoid accumulation by provoking growth, pigmentation, photosynthesis, and secondary metabolites production of cannabis grown in an indoor environment. After three weeks of vegetative growth under greenhouse condition, plants were further grown for 90 days in a plant factory treated with 4 LED light compositions with a canopy-level photosynthetic photon flux density (PPFD) of 300 µmol m^−2^ s^−1^ for 16 h. Photosynthetic pigments and photosynthetic rate were linearly increased up to 60 days and then sharply decreased which was found most prominent in L3: MB 240 (Red 85% + Blue 15%) and L4: PF 240 (Red 70% + Blue 30%) LED light compositions. A high concentration of H_2_O_2_ was also observed in L3 and L4 treatments which provoked lipid peroxidation in later growth stage. In addition, higher accumulation of cannabinoid was observed under L4 treatment in most cases. It is also evident that higher ROS created a cellular stress in plant as indicated by higher osmolyte synthesis and enzyme activity which initiate quick maturation along with higher cannabinoids accumulation in cannabis plant. Therefore, it can be concluded that ROS metabolism has a crucial role in morpho-physiological acclimation and cannabinoid accumulation in hemp plants. The findings of this study provide further insight on the use of LED light to maximize the production of cannabinoid.

## 1 Introduction

Light is the unique source of energy for photosynthesis and acts as a driving force for plant growth. The quality, quantity and duration of light are useful input materials that regulate not only the growth and development of plants but also ensure their sustainable production under environmental conditions (Jenkins and Livesay, 2021). Growing plants under artificial light allow growers to maintain horticultural traits, including plant morphology, growth habits, flowering, quality and endpoint plant productivity (Rodriguez-Morrison et al., 2021). However, comprehensive knowledge regarding the light requirements of various plant species for optimum growth and the impact of light intensity as well as spectral composition on plant metabolism and nutritional status is still insufficient (Monostori et al., 2018). Cannabis (*Cannabis sativa* L.) is a high-value crop capable of growing in a controlled environment profitably under artificial light, especially light-emitting diode (LED) technology, to ensure high photon output cost-effectively depending on the choice of LED and drive current (Kusuma et al., 2020; Westmoreland et al., 2021). The high light intensity with proper photoperiod is needed during the vegetative growth stage to maximize cannabis growth and to initiate the budding (Arnold, 2013). In this context, the quality of LED had a significant impact on cannabis production as LED fixtures can be made with unique spectra that have the potential to increase the quality and targetted yield (Magagnini et al., 2018; Westmoreland et al., 2021). Moreover, a considerable change in shoot architecture, inflorescence mass and the alteration in the content of cannabinoids, terpenes, and other bioactive properties of the plant extracts may significantly vary on LED light composition (Namdar et al., 2019).

Light systems for cannabis cultivation need to ensure quality and quantity to maximize plant productivity and achieve a high level of secondary metabolite production (Jenkins and Livesay, 2021). The quality and intensity of light should consider the physiological and photosynthetic enhancement to ensure maximum, uniform, and consistent productivity with minimum deleterious effects of high leaf temperature (Greer et al., 1986) and photooxidative damage (Demmig-Adams and Adams Iii, 1992). Although cannabis plants are well known for a high degree of plasticity concerning the light spectrum intensity, evidence proved that compatible spectra could maintain leaf temperature within an optimal range to ensure maximum photosynthetic rate (Jenkins and Livesay, 2021). For this reason, understanding the spectral quality in photosynthesis is critical when selecting a lighting system with proper light quality and quantity for any indoor cultivation.

Cannabis produces unique secondary metabolites called cannabinoid containing alkylresorcinol and monoterpene groups that have a tremendous interest due to their pharmacological activities, such as psychoactive and analgesic effects (Mechoulam, 1970; Shoyama et al., 2008). For this reason, cannabinoid have attracted a great deal of attention, whereas LED light combination that produces higher metabolites demands more research from a different perspective. Moreover, in recent studies, cannabis plants produced higher secondary metabolites under abiotic stress conditions (Caplan et al., 2019), and such stress has a direct connection with reactive oxygen species (ROS) network (You and Chan, 2015; Choudhury et al., 2017). ROS are the molecules that include highly reactive free radicals (e.g., *superoxide anion*[ $O_{2}^{•-}$ ] and stable non-radical oxidants [e.g., *hydrogen peroxide* (H_2_O_2_)] produced in plant cells during normal metabolic processes (Mittler et al., 2004). $O_{2}^{•-}$ is rapidly converted to H_2_O_2_ in the cell by the activities of the antioxidant enzyme system. H_2_O_2_ and $O_{2}^{•-}$ have attracted the main focus of ROS biology in recent years; among them, H_2_O_2_ not only play the role of ROS but also can act as an intercellular signalling molecule regulating plant growth and development (Gough and Cotter, 2011; Hossain et al., 2015; Černý et al., 2018; Huang et al., 2019). Therefore, the objectives of this study were to examine the effects of light-spectral quality on growth-related morpho-physiological traits of cannabis and cannabinoid content and also disclose their relationship with ROS metabolism.

## 2 Materials and methods

### 2.1 Seedling growth condition

The alpine star CBD feminized hemp seeds (*Cannabis sativa* L. strain KHV 1) were collected from the weed seed express, Haarlem, Netherlands and sown in sixteen cells plug tray (27 cm× 27 cm × 6 cm) filled with commercial soil mixture (Bio-soil No. 1, Heungnong Agricultural Materials Mart, Korea) in a glasshouse. Before sowing, the seeds were surface sterilized [70% (v/v) ethanol, 0.1% (w/v) HgCl_2_ and 0.2% (w/v) thiram] and soaked in water for 24 h at room temperature to facilitate the germination. The environmental conditions such as temperature, relative humidity (RH), and photoperiod were recorded at 30/25°C (day/night), 60%–70%, and 12 h, respectively. The seedlings were irrigated daily using tap water to the field capacity level. After three weeks of growth, the seedlings were transferred to the plant factory for treating under 4 LED lights. The nutrient element concentration (g L^-1^) in the system was: Ca (NO_3_). 4H_2_O, 15; KNO_3_, 37.9; (NH_4_)_2_HPO_4_, 37.9; MgSO_4_, 16; K_2_SO_4_, 43; Fe EDTA, 4.6; MnSO_4_, 0.308; H_3_BO_3_, 0.572; ZnSO_4_, 0.036; CuSO_4_, 13; (NH_4_)_6_Mo_7_O_24_.4H_2_O, 0.004. The E.C. and pH ranges were adjusted to 1.5-1.7 (dS m^-2^) and 5.8-6.0, respectively.

### 2.2 Light treatment

**Figure 1:**
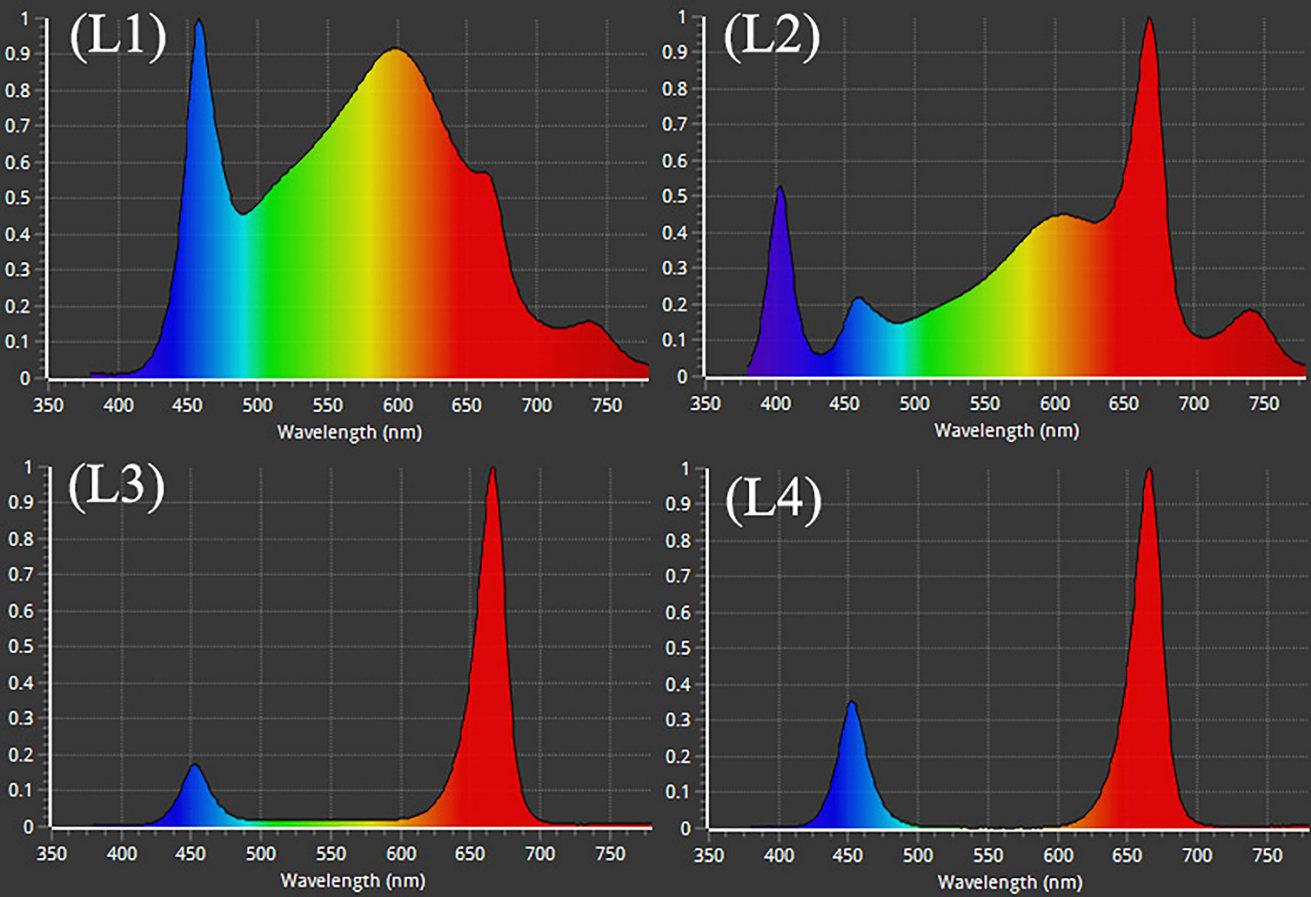
Different LED light used in the study. L1, MW 240 (Red 35% + Blue 25% + Green 40%); L2, FS 240-UV (Red 40% + UVA and Blue 26% + Green 29% + Far red 5%); L3, MB 240 (Red 85% + Blue 15%); L4, PF 240 (Red 70% + Blue 30%).

After adjustment, the plants were subjected to treatment with 4 LED lights (Bisol LED light Co., Seoul, Korea). The LED light treatments were L1: MW 240 (Red 35% + Blue 25% + Green 40%), L2: FS 240 UV [Red 40% + (Blue + UV-A 26%) + Green 29% + Far-red 5%], L3: MB 240 (Red 85% + Blue 15%), and L4: PF 240 (Red 70% + Blue 30%) (**Figure 1**). The photosynthetic photon flux density (PPFD), photoperiod, and temperature of the chamber were 300 µmol m^−2^ s^−1^, 16 h (6.00 AM to 10.00 PM), and 23 to 27°C, respectively. The PPFD and percent of lights were checked and adjusted at the top leaf level every other day by a PG200N spectral PAR meter (UPRtek, Zhunan township, Taiwan). The plant factory was designed for an automatic system where nutrient formulated water was injected into the plant root zone (growing pot) for twenty seconds every two minutes. Data were collected at 30, 60, and 90 days after treatment (DAT). After 90 DAT, the plants under L3 and L4 light treatments were died. For this reason, few of the biochemical and morphological parameters were omitted under L3 and L4 treatments.

### 2.3 Determination of the morphological traits of hemp seedlings

Three samples (three plants/sample) from each light treatment were randomly selected at the end of the treatment to determine the shoot length (SL), number of branches (BN), leaf length (LL), and leaf width (LW). The third leaf from the top was selected for measuring the length and width of the leaves.

### 2.4 Photosynthetic pigments analysis

For the determination of photosynthetic pigments, the freeze-dried (25 mg) leaves were extracted (10 mL of 80% acetone) and placed at room temperature for 15 min, then centrifuged at 4000 rpm for 10 min. The absorbance was taken at 663, 647, and 470 nm using a spectrophotometer (UV-1800 240 V, Shimadzu Corporation, Kyoto, Japan). Chlorophyll *a*, Chlorophyll *b*, and Car were determined according to the formula proposed by Lichtenthaler (1987) and expressed as mg g^−1^ DW:


$$\text{Chl }a=\;12.25\;\times {\text{ A}}_{663}-\;2.79\;\times {\text{ A}}_{647}$$

$$\text{Chl }b=\;21.50\;\times {\text{ A}}_{647}-\;5.10\;\times {\text{ A}}_{663}$$

$$Car\;=\;\left[\text{(1000}\times {\text{A}}_{470}\text{) }-\text{ (}1.82\times \text{Chla) }-(85.02\times \text{Chlb)}\right]/198$$

### 2.5 Leaf gas exchange measurement

The net photosynthetic rate (A, µmol m^−2^ s^−1^), transpiration rate (E, mmol m^−2^ s^−1^), and stomatal conductance (gs, mmol m^−2^ s^−1^) were measured on well-developed leaves (3rd node from the top) of four samples under each treatment using an LCpro gas analyzer (ADC BioScientific Ltd., Hoddesdon, Herts EN11 ONT, UK). The levels of A, gs, and E were measured at the ambient CO_2_ concentration, air temperature at 25-26 °C and (PPFD, 1000 µmol mol^-1^S^-1^) in the leaf chamber. The measurements of gas exchange were carried out at the mid-day between 10.00 AM and 3.00 PM.

### 2.6 Determination of photosystem II quantum yield

The photosynthetic quantum yield (Fv/Fm) of photosystem II (PSII) was measured using a Fluor Pen FP 100 (Photon system Instruments, Drasov 470, 66424 Drasov, Czech Republic) under the dark-adapted condition at least for 20 min.

### 2.7 Determination of malondialdehyde (MDA) and H_2_O_2_ content

Malondialdehyde (MDA) was measured to determine the lipid peroxidation in the hemp leaves. For MDA assay, 200 mg fresh leaf sample was ground in 5 mL of 0.1% trichloroacetic acid and centrifuged at 10,000× *g* for 10 min at 4°C. A 4 mL of 20% trichloroacetic acid (TCA) containing 0.5% thiobarbituric acid was added to 1 mL of supernatant. The mixture was heated at 95°C for 30 min, followed by cooled quickly on an ice bath. The resulting mixture was centrifuged again at 5000 rpm for 15 min, and the absorbance was taken at 532 nm and 600 nm. An extinction coefficient of 155 mM^−1^ cm^−1^ was used to calculate the MDA concentration (Heath and Packer, 1968). The H_2_O_2_ content was estimated according to the method developed by Singh et al. (2006). 200 mg of fresh leaf sample was extracted in 5 mL of 0.1% (*w/v*) TCA and centrifuged at 12,000× *g* for 15 min in a refrigerated centrifuge. Then 0.5 mL of the supernatant was added to 0.5 mL of 10 mM potassium phosphate buffer (pH 7.0). After that, 1 mL of 1 M KI was added to the mixture and placed in a dark place (1 h) for incubation. The absorbance was measured at 390 nm, where a standard H_2_O_2_ curve was prepared to calculate the concentration of H_2_O_2_ in the sample.

### 2.8 Activities of antioxidant enzymes

For the analysis of antioxidant enzymes, leaf samples were collected and immersed immediately in liquid nitrogen and stored at −80°C until use. A 200 mg sample was homogenized in 5 mL of 50 mM sodium phosphate buffer solution (pH 7.8) using a pre-chilled mortar and pestle, then centrifuged at 15,000× *g* for 20 min at 4°C. After collecting the supernatant, the enzyme extract was stored at 4°C for analysis (Islam et al., 2021a).

The superoxide dismutase activity (SOD; EC 1.15.1.1) was estimated by the method described earlier (Islam et al., 2020). The reaction mixture for estimating SOD contained 50 mM sodium phosphate buffer with 0.1 mM EDTA, 12 mM methionine, 75 µM NBT, and 50 mM Na_2_CO_3_. Then, a 100 µL enzyme extract or 100 µL buffer was used in the sample or blank, respectively. After that, 300 µL of 0.1 mM Riboflavin was added to the reaction mixture to make 2 mL of the final volume. The tubes were shaken and irradiated under the fluorescent light (15 W) for 15 min. The absorbance was taken at 560 nm by a spectrophotometer. From the result, 50% inhibition of NBT reduction was considered as one unit of the enzyme (Giannopolitis and Ries, 1977).

The activities of guaiacol peroxidase (POD; EC 1.11.1.7) and catalase (CAT; EC 1.11.1.6) were assayed by the method as described by (Zhang, 1992). For POD assay, a 3 mL reaction mixture contained 100 µL enzyme extract, 100 µL guaiacol (1.5%, *v/v*), 100 µL H_2_O_2_ (300 mM), and 2.7 mL 25 mM sodium phosphate buffer with 2 mM EDTA (pH 7.0). The absorbance was measured by a spectrophotometer at 470 nm (ε = 26.6 mM cm^−1^). On the other hand, the assay mixture for CAT contained 100 µL of enzyme extract, 100 µL of H_2_O_2_ (300 mM), and 2.8 mL of 50 mM phosphate buffer with 2 mM EDTA (pH 7.0). The decreased absorbance rate was measured at 240 nm (ε = 39.4 mM cm^−1^).

### 2.9 Determination of tetrahydrocannabinol (THC), tetrahydrocannabinolic acid (THCA), cannabidiol (CBD), and cannabidiolic acid (CBDA)

The freeze-dried (100 mg) leaf sample was dissolved in 5 mL of methanol (100%) and sonicated at room temperature for 20 min. After filtration through a syringe filter (0.45 µM, Millipore, Bedford, MA, USA), the solution was kept in a refrigerator at 4 °C. The HPLC system (Shimadzu LC-20 AT, Shimadzu Co., Ltd., Kyoto, Japan) with a UV-VIS detector and a reverse phase Zorbax SB-C18 column (4.6 mm × 100 mm, 3.5 µm, Agilent Technologies, Inc., Santa Clara, CA, USA) was used. The mobile phase was 70% acetonitrile containing 0.1% phosphoric acid with isocratic elution mode. The retention times of standard CBDA, CBD, Δ9-THC, and Δ9-THCA were 3.60, 4.34, 9.60, and 13.00 min, respectively. A 10 µL sample was injected where the flow rate and oven temperature were 1.5 mL min^−1^ and 27°C, respectively. The detection wavelength was used 275 nm with three biological replications.

### 2.10 Statistical analysis

All results were expressed as mean ± SEM. The data were analyzed using SAS 9.4 (SAS Institute Inc., Cary, NC, USA). A one-way analysis of variance among the light treatments within each time for all graphs, while a two-way analysis of variance among the light treatments and time of observations were done for the table. The mean differences were compared by Tukey’s *post-hoc* multiple comparison test. P values<0.05 were considered to be significant. The principal component analysis (PCA) was carried out using OriginLab 10.0 (OriginLab, Northampton, MA, USA).

## 3 Results

### 3.1 Effect of LED light on the morphological traits of hemp seedlings

**Figure 2:**
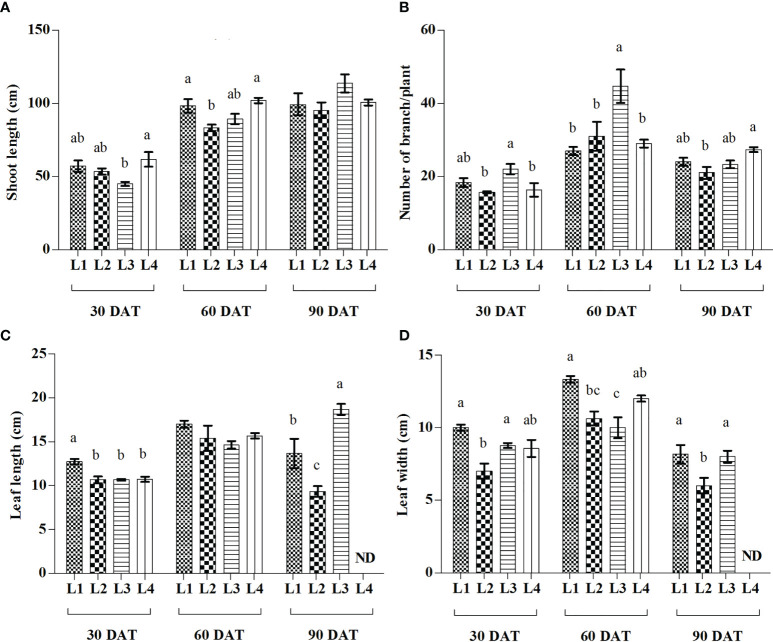
Effect of LED spectra on the shoot length **(A)**, number of branch **(B)**, leaf length **(C)**, and leaf width **(D)** of hemp seedlings at 30, 60, and 90 DAT. Here and subsequent figures: L1, MW 240 (Red 35% + Blue 25% + Green 40%); L2, FS 240-UV (Red 40% + UVA and Blue 26% + Green 29% + Far red 5%); L3, MB 240 (Red 85% + Blue 15%); L4, PF 240 (Red 70% + Blue 30%); ND, not detected. All treatments used a photosynthetic photon flux density of 300 µmol m^−2^ s^−1^. Column height indicates the mean, vertical bars indicate the standard error of the mean (n = 4), and different letters indicate significant differences at p < 0.05.

From the current study, it was observed that different LED treatments and observation times have a significant effect on SL, BN, LL, and LW of hemp seedlings (**Figure 2**). At 90 DAT, the maximum increase of SL was recorded in L3 (153.53%) treatment followed by L2 (78.75%), whereas higher proliferation of BN was manifested by L2 (97.95%) followed by L4 (77.59%) at 60 DAT, compared to 30 DAT. On the other hand, the maximum increment of LL was recorded in L3 (75%) treatment at 90 DAT followed by L4 (45.94%) at 60 DAT, whereas L2 (51.86%) followed by L4 (40.19%) produced higher LW at 60 DAT, compared to 30 DAT.

### 3.2 Effect of LED light on the photosynthetic pigments of hemp seedlings

**Figure 3:**
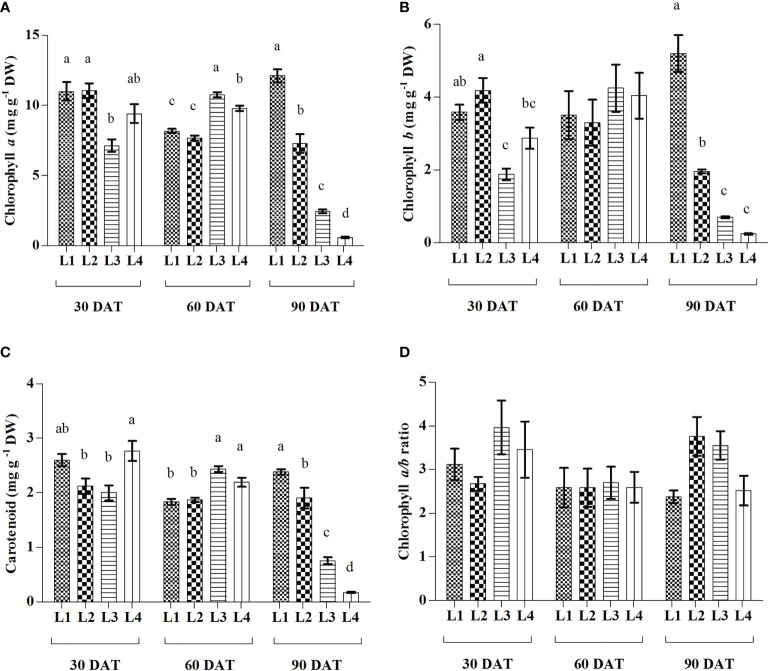
Effect of LED spectra on Chlorophyll a **(A)**, Chlorophyll b **(B)**, Carotenoid **(C)**, and Chlorophyll a/b ratio **(D)** of hemp seedlings at 30, 60, and 90 DAT. Here and subsequent figures: L1, MW 240 (Red 35% + Blue 25% + Green 40%); L2, FS 240-UV (Red 40% + UVA and Blue 26% + Green 29% + Far red 5%); L3, MB 240 (Red 85% + Blue 15%); L4, PF 240 (Red 70% + Blue 30%). All treatments used a photosynthetic photon flux density of 300 µmol m^−2^ s^−1^. Column height indicates the mean, vertical bars indicate the standard error of the mean (n = 4), and different letters indicate significant differences at p < 0.05.

In comparison to 30 DAT, higher increment of both Chl *a* and Chl *b* were observed in L3 (50.91% and 127.27%) treatment at 60 DAT followed by L1 (10.19% and 44.56%) at 90 DAT and L4 (4.14% and 40.76%) at 60 DAT (**Figure 3**). In case of Car, the maximum increment was recorded in L3 (22.11%) treatment at 60 DAT. On the other hand, no significant differences were observed in Chl a/b ratio under any treatment at 3 observation times, but L2 (40.44%) showed highest increment at 90 DAT. Results also showed that L3 and L4 treatments manifested maximum reduction of Chl *a* (65.77% and 93.72%), Chl *b* (63.10% and 91.63%) and Car (62.06% and 93.80%) at 90 DAT compared to 30 DAT.

### 3.3 Effect of LED light on the photosynthetic gas exchange and maximum quantum yield of hemp seedlings

**Figure 4:**
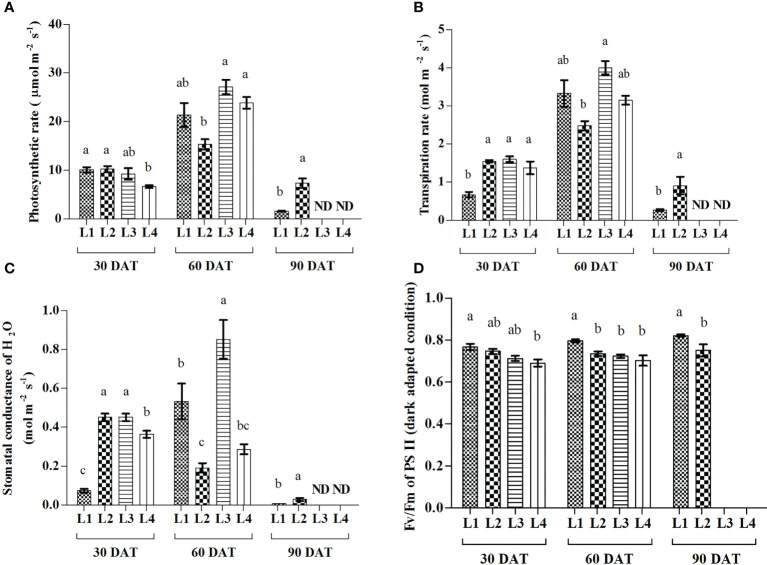
Effect of LED spectra on photosynthetic rate **(A)**, transpiration rate **(B)**, stomatal conductance **(C)**, and maximum photosynthetic efficiency of PS II **(D)** of hemp seedlings at 30, 60, and 90 DAT. Here and subsequent figures: L1, MW 240 (Red 35% + Blue 25% + Green 40%); L2, FS 240-UV (Red 40% + UVA and Blue 26% + Green 29% + Far red 5%); L3, MB 240 (Red 85% + Blue 15%); L4, PF 240 (Red 70% + Blue 30%); ND, not detected. All treatments used a photosynthetic photon flux density of 300 µmol m^−2^ s^−1^. Column height indicates the mean, vertical bars indicate the standard error of the mean (n = 4), and different letters indicate significant differences at p < 0.05.

Results showed that L3 and L4 treatments manifested the maximum increase of A (193.61% and 259.58%) at 60 DAT compared to 30 DAT, whereas the maximum increase of E (397%, 149.4% and 129.9%) and gs (657%, 88.9% and 19.44%) were recorded in L1, L3 and L4 treatments (**Figure 4**). On the other hand, the maximum increase of quantum yield was recorded in L1 (7.89%) and L3 (4.22%) treatments at 90 and 60 DAT, respectively.

### 3.4 Influence of LED on lipid peroxidation and hydrogen peroxide

**Figure 5:**
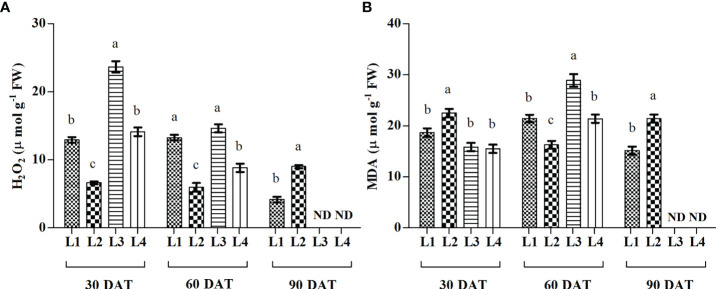
Effect of LED spectra on hydrogen peroxide (**A**, H_2_O_2_) and malondialdehyde (**B**, MDA) of hemp seedlings at 30, 60, and 90 DAT. Here and subsequent figures: L1, MW 240 (Red 35% + Blue 25% + Green 40%); L2, FS 240-UV (Red 40% + UVA and Blue 26% + Green 29% + Far red 5%); L3, MB 240 (Red 85% + Blue 15%); L4, PF 240 (Red 70% + Blue 30%); ND, not detected. All treatments used a photosynthetic photon flux density of 300 µmol m^−2^ s^−1^. Column height indicates the mean, vertical bars indicate the standard error of the mean (n = 4), and different letters indicate significant differences at p < 0.05.

The concentration of H_2_O_2_ was recorded higher in L3 and L4 treatments at 30 DAT (23.15 µmol g^-1^ FW and 14.12 µmol g^-1^ FW), and it decreased by 38.2% and 37.6% at 60 DAT compared to 30 DAT (**Figure 5**). On the other hand, the maximum increase of MDA was found in L3 (82.34%) and L4 (38.09%) treatments at 60 DAT.

### 3.5 Effect of LED light on osmolytes and secondary metabolites

Higher increase in proline, ascorbic acid, sucrose and TFC concentrations were observed at 60 DAT in L3 treatment by 242.4%, 131.96%, 12.85% and 7.42%, respectively, compared to 30 DAT (**Table 1**). On the other hand, the concentration of TSC increased maximum in L1 (44.38%) treatment at 60 DAT, whereas TPC increased maximum in L4 (28.87%) treatment at 90 DAT.

**Table 1:** Effect of LED spectra on proline, ascorbic acid, total soluble carbohydrate (TSC), sucrose, total phenolic content (TPC) and total flavonoid content (TFC) of hemp seedlings at 30, 60, and 90 DAT.

	Treatments	Proline (µmoles/g DW)	Ascorbic acid (mg/g DW)	TSC (mg/g DW)	Sucrose (mg/g DW)	TPC (mg/g DW)	TFC (mg/g DW)
30 DAT	L1	27.53 ± 2.83^gh^	11.66 ± 0.45^def^	65.59 ± 7.05^c^	169.10 ± 2.55^e^	0.78 ± 0.002^d^	6.14 ± 0.05^b^
	L2	14.35 ± 2.62^i^	15.43 ± 0.25^bcd^	71.56 ± 0.15^bc^	191.99 ± 1.00^c^	0.67 ± 0.003^gh^	4.73 ± 0.02^d^
	L3	24.65 ± 2.32^h^	9.01 ± 1.68^fg^	69.88 ± 5.08^bc^	177.37 ± 0.67^d^	0.83 ± 0.004^b^	6.54 ± 0.02^b^
	L4	35.57 ± 2.83^fg^	18.51 ± 1.59^ab^	74.77 ± 1.72^bc^	211.03 ± 0.67^a^	0.79 ± 0.002^d^	5.47 ± 0.01^c^
60 DAT	L1	68.15 ± 1.62^c^	15.25 ± 1.12^bcd^	84.17 ± 1.48^ab^	169.87 ± 3.00^e^	0.66 ± 0.001^h^	5.06 ± 0.03^cd^
	L2	66.16 ± 1.53^c^	17.16 ± 0.30^abc^	91.47 ± 2.24^a^	201.12 ± 0.17^b^	0.68 ± 0.003^g^	4.89 ± 0.02^d^
	L3	84.40 ± 1.45^b^	20.89 ± 1.58^a^	89.82 ± 1.24^a^	200.16 ± 1.83^b^	0.72 ± 0.004^e^	7.02 ± 0.03^a^
	L4	45.82 ± 1.70^e^	19.97 ± 0.39^a^	92.98 ± 1.16^a^	184.87 ± 0.22^c^	0.81 ± 0.003^c^	5.47 ± 0.02^c^
90 DAT	L1	55.61 ± 0.83^d^	20.24 ± 1.15^a^	94.70 ± 0.19^a^	188.52 ± 1.55^c^	0.62 ± 0.001^i^	4.15 ± 0.02^e^
	L2	35.95 ± 0.89^fg^	14.20 ± 0.25^cde^	73.51 ± 0.34^bc^	139.10 ± 0.89^f^	0.70 ± 0.004^f^	4.16 ± 0.02^e^
	L3	42.33 ± 0.96^ef^	10.57 ± 0.94^ef^	67.46 ± 3.98^c^	85.64 ± 1.11^h^	0.70 ± 0.004^f^	3.36 ± 0.02^f^
	L4	104.57 ± 0.77^a^	6.07 ± 1.03^g^	82.97 ± 1.95^ab^	101.51 ± 1.17^g^	1.02 ± 0.004^a^	3.62 ± 0.03^f^

Different letters show significant differences at P<0.05. Values are expressed as mean ± SEM.

### 3.6 Effect of LED light on antioxidant enzymes activities

**Figure 6:**
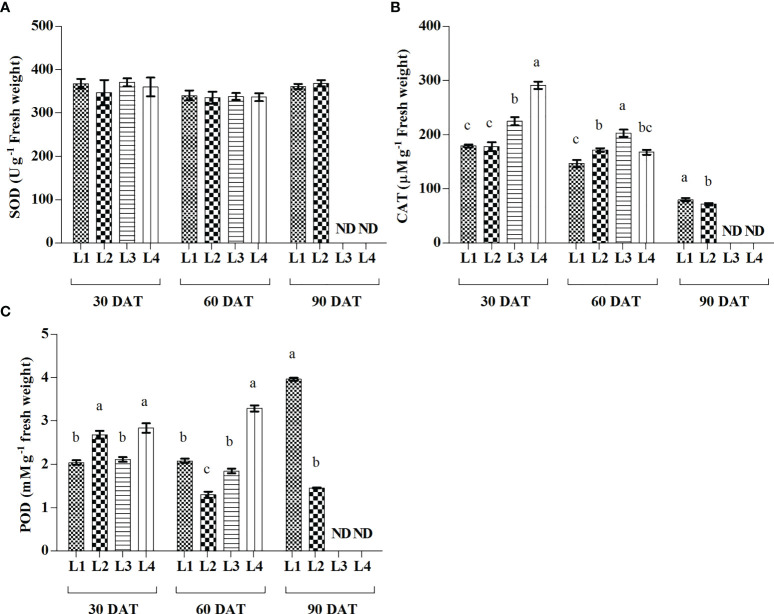
Effect of LED spectra on superoxide dismutase (SOD) **(A)**, catalase (CAT) **(B)**, and guaiacol peroxidase (POD) **(C)** of hemp seedlings at 30, 60, and 90 DAT. Here and subsequent figures: L1, MW 240 (Red 35% + Blue 25% + Green 40%); L2, FS 240-UV (Red 40% + UVA and Blue 26% + Green 29% + Far red 5%); L3, MB 240 (Red 85% + Blue 15%); L4, PF 240 (Red 70% + Blue 30%); ND, not detected. All treatments used a photosynthetic photon flux density of 300 µmol m^−2^ s^−1^. Column height indicates the mean, vertical bars indicate the standard error of the mean (n = 4), and different letters indicate significant differences at p < 0.05.

From the results, plants attained significantly higher activity of CAT in the treatment L4 (291.45 µmolg^-1^ FW) treatment followed by L3 (224 µmolg^-1^ FW) treatment at 30 DAT, while it was rapidly decreased by 42.47% and 9.82%, respectively at 60 DAT (**Figure 6**). POD activity increased maximum in L1 and L4 treatments at 90 DAT and 60 DAT, respectively. On the other hand, no significant change was observed in case of SOD activity at any level of observation.

### 3.7 Effect of LED light on cannabinoid accumulation

**Figure 7:**
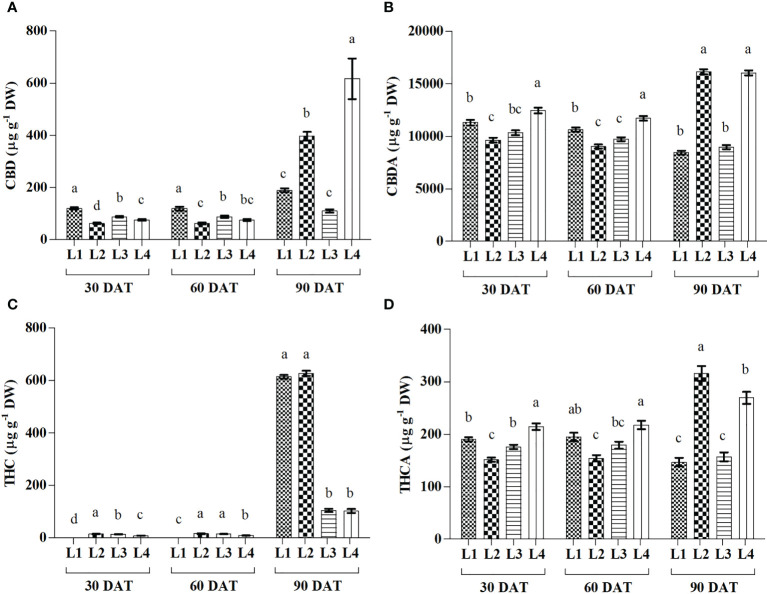
Effect of LED spectra on cannabidiol (CBD) **(A)**, cannabidiolicacid (CBDA) **(B)**, tetrahydrocannabinol (THC) **(C)** and tetrahydrocannabinolic acid (THCA) **(D)** of hemp seedlings at 30, 60, and 90 DAT. Here and subsequent figures: L1, MW 240 (Red 35% + Blue 25% + Green 40%); L2, FS 240-UV (Red 40% + UVA and Blue 26% + Green 29% + Far red 5%); L3, MB 240 (Red 85% + Blue 15%); L4, PF 240 (Red 70% + Blue 30%); ND, not detected. All treatments used a photosynthetic photon flux density of 300 µmol m^−2^ s^−1^. Column height indicates the mean, vertical bars indicate the standard error of the mean (n = 4), and different letters indicate significant differences at p < 0.05.

Significant variations in Cannabidiol (CBD), Cannabidiolic acid (CBDA), Tetrahydrocannabinol (THC), and Tetrahydrocannabinolic acid (THCA) were observed under 4 LED light at 3 observation times (**Figure 7**). The maximum increase in CBD and CBDA was recorded in L2 (526.81% and 67%) and L4 (708.57% and 28.73%) treatments at 90 DAT. Results also showed that THC increased by 672.29% and 1090.14% in L3 and L4 treatments, respectively, at 90 DAT, compared to 30 DAT, which is lower than other treatments. On the other hand, the maximum increase in THCA was recorded by L2 (108.32%) treatment at 90 DAT as compared to 30 DAT.

### 3.8 The PCA analysis unveiled the connection between variables and treatments

**Figure 8:**
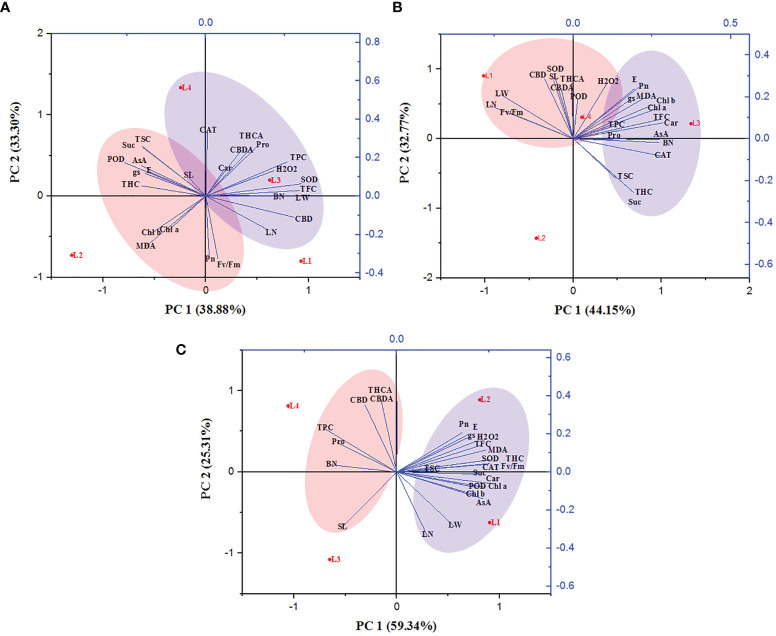
Principal component analysis of hemp seedlings under four LED light sources at 30 **(A)**, 60 **(B)** and 90 **(C)** DAT. The lines starting from the central point of the biplots display negative or positive associations of different variables, and their proximity specifies the degree of correlation with a specific treatment. L1, MW 240 (Red 35% + Blue 25% + Green 40%); L2, FS 240-UV (Red 40% + UVA and Blue 26% + Green 29% + Far red 5%); L3, MB 240 (Red 85% + Blue 15%); L4, PF 240 (Red 70% + Blue 30%). SL, shoot length; BN, branch number; LN, leaf length; LW, leaf width; Chl a, chlorophyll a; Chl b, chlorophyll b; Car, carotenoid; Pn, photosynthetic rate; E, transpiration rate; gs, stomatal conductance; Fv/Fm, maximum photosynthetic efficiency of PS II; H_2_O_2_, hydrogen peroxide; MDA, malondialdehyde; Pro, proline; AsA, ascorbic acid; TSC, total soluble carbohydrate; Suc, sucrose; TPC, total polyphenol content; TFC, total flavonoid content; SOD, superoxide dismutase; CAT, catalase; POD, guaiacol peroxidase; THC, tetrahydrocannabinol; THCA, tetrahydrocannabinolic acid; CBD, cannabidiol; CBDA, cannabidiolic acid.

The correlation between the growth variables, photosynthetic traits, biochemical attributes, and secondary metabolites of hemp plants was ascertained. The entire experimental data were subjected to a principal component analysis based on the clustering method (**Figure 8**). The PCA loading plot revealed that PCA 1 and PCA 2 accounted for 72.18%, 76.92%, and 84.65% of total variation among the studied parameters at 30, 60, and 90 DAT, respectively. At 30 DAT, H_2_O_2_ was positively correlated with Pro, Car, SOD, CAT, TPC, TFC, THCA and CBDA, and clustered with the treatment L3 and L4 while MDA manifested a negative correlation with them. In case of 60 DAT, both H_2_O_2_ and MDA clustered with the treatments L3 and L4 while they are strongly correlated with Pro, AsA, TPC, TFC, SOD, CAT, POD, THCA, CBDA and CBD, indicating that both L3 and L4 treatments have a prominent role in stress generation in hemp plants. Similarly, L4 treatment also maintained a positive correlation with Pro, TPC, THCA, CBDA and CBD.

## 4 Discussion

**Figure 9:**
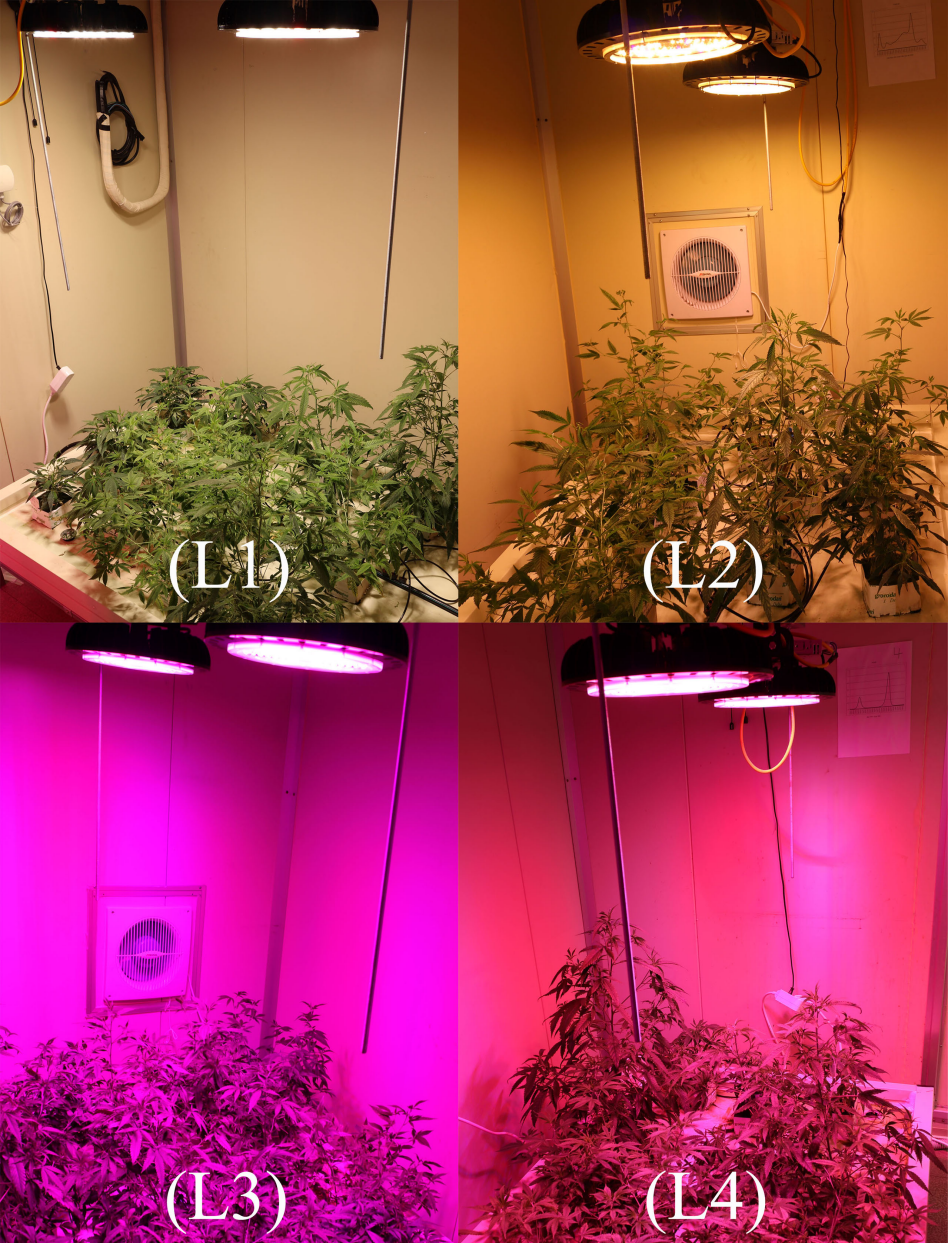
Effect of LED light on growth and morphological traits of hemp plants at 30 DAT. L1, MW 240 (Red 35% + Blue 25% + Green 40%); L2, FS 240-UV (Red 40% + UVA and Blue 26% + Green 29% + Far red 5%); L3, MB 240 (Red 85% + Blue 15%); L4, PF 240 (Red 70% + Blue 30%).

Light is an essential environmental factor that affects plant growth and development, and plants respond to light variations to complete their life cycle. The light-emitting diode (LED) is an energy-efficient and rapidly developing lighting technology used widely nowadays. Irradiance with various spectral range and their combinations lead to a change in plant photosynthesis that ultimately plays a crucial role in plant establishment and the composition of secondary metabolites (Wei et al., 2021). From the results of the present study, a higher growth rate was observed in L3 (153.5%) and L2 (78.75%) at 90 DAT compared to 30 DAT. Similarly, higher increase of BN was recorded in L3 (103%) treatment and L2 (97.86%) at 60 DAT. On the other hand, both LL and LW were found higher at 60 DAT in L2 (44.36% and 51.89%) and L4 (45.98% and 40%) treatments. In the treatment L2, despite having higher increment rate, hemp attained comparative lower value of morphological characteristics where an additional FR light was used along with others. Similar results were also described in a previous study, where the addition of FR to R and B decreased the LL and SL of tomato plants (Kalaitzoglou et al., 2019). On the other hand, comparative higher SL, BN, LL, and LW were recorded in L1, L3 and L4 treatments in most cases. In general, plants grown under red light show higher shoot length than blue (Rabara et al., 2017), which might be due to the high sensitivity of phytochrome to red and far-red light, and to a lesser extent, blue light (Eichhorn Bilodeau et al., 2019). Although, green light is considered less effective for plant growth since plant photosynthetic pigments have limited absorbance for these wavelengths. However, there is evidence that a low percentage of green light influences plant morphology, including leaf growth, stomatal conductance, and early stem elongation (Kim et al., 2005). Besides, blue and UV-A trigger cryptochrome and phototropin that regulate chloroplast relocation, elongation, stomatal opening and photosynthesis (Schwartz and Zeiger, 1984). These hypotheses support our findings as L3 and L4 treatments consist of a high percentage of red light, whereas L1 consists of a combination of red, blue, and green light (**Figure 9**).

The chlorophyll measurement is a reliable tool that is commonly used as evidence of plant growth and vigour, where its concentration has a high correlation with photosynthesis mechanisms (Rabara et al., 2017). In the present study, the treatment L3 consisted of a relatively lower percentage of blue light that influenced the low accumulation of Chl *a*, Chl *b*, and Car at 30 and 90 DAT, while it influenced the higher accumulation of all pigments at 60 DAT. These results indicate a shorter life cycle and early senescing or chlorosis by the effect of light treatment on plants (**Figure 3**). Similar results were also observed in L4 treatment where plants gradually decreased all pigments compared to others. Opposite results were observed in L1 treatment where plants accumulate higher pigments at 30 DAT and tend to decrease at 60 DAT. However, plants accumulated a maximum concentration of pigments at 90 DAT. Previous results obtained in *Tripterospermum japonicum* and *Lippiaalba* showed that chlorophyll accumulation was influenced by a mixture of red and blue light (Moon et al., 2006; Batista et al., 2016). Besides, the addition of low-energy far-red light to the growth spectrum along with the high proportion of blue light may increase photosynthetic pigment concentration and gas exchange (Kong and Nemali, 2021). UV-A radiation was also described to enhance photosynthetic pigments in lettuce (Chen et al., 2019). Despite low absorbance, green light penetrates deeper and excites chlorophyll, and at high PPFD, it may achieve higher photosynthetic efficiency due to the uniform absorption throughout leaves (Liu and van Iersel., 2021). Another study on lettuce described that photosynthetic pigments, absorption of PPFD, and CO_2_ assimilation ratio showed a remarkable decrease under the LED spectra lacking green light when compared to a broad spectrum of LED light (Liu et al., 2017). These results comply with our findings as L1 and L2 treatments showed higher pigments accumulation, where green, UV-A, and FR were used as supplementary with red and green.

Photosynthesis plays an important role in plant growth and development due to its direct connection with productivity in a given environment (Eichhorn Bilodeau et al., 2019). Both photosynthetic rate and transpiration rate rapidly increased in L3 and L4 treatments at 60 DAT compared to 30 DAT, and plants died at 90 DAT (**Figure 4**). A similar observation was also observed in plant growth and pigments accumulation. From these results, it is plausible that plants grown under red and blue spectra achieved quick maturity with a short life cycle. Photosynthesis occurs within the chloroplast of palisade and spongy mesophyll cells in epidermal layers of leaves (Mishra, 2004). The higher mass and photosynthetic pigment levels of plants grown under red/blue LED light indicated that there is greater use of light in these regions of the visible spectrum (Batista et al., 2016). In general, a higher photosynthetic rate can be achieved under red light, while blue light induces the strongest preferential excitation of PS II (Hogewoning et al., 2012; Kalaji et al., 2014). Besides, a high rate of leaf abscission was assumed (Batista et al., 2016) that occurred in plants grown under red/blue light, which also modified the timing of some stress responses in plants (Hoffman et al., 2015).

Light wavelength and intensity that provokes photosynthesis and photomorphogenesis are widely used to quantify the light in the experiment related to plant-light interaction (Eichhorn et al., 2019; Olle and Viršile, 2013; Singh et al., 2015). Each spectral band of light can induce specific mechanisms and responses in the plant, affecting subsequent plant responses to stress (Bayat et al., 2018). Due to the high sensitivity to the spectral distribution, plants perceive the change in light spectra and intensity through several protein photoreceptors (Fankhauser and Chory, 1997). In our study, H_2_O_2_ accumulation was detected following LED spectral stress. Generally, excess light energy beyond photosynthetic capacity induces ROS (Pospíšil, 2016), where chloroplasts and peroxisomes act as leading ROS producers in plants (Choudhury et al., 2013). In the present experiment, L3 and L4 treatments accumulated higher concentrations of H_2_O_2_ and MDA at 30 and 60 DAT, as also evident by PCA analysis (**Figure 8**). Comparative lower accumulation of H_2_O_2_ (6.63 µmol g^-1^ FW) and MDA (16.27 µmol g^-1^ FW) were observed in L2 at 60 DAT, and based on it, L3 and L4 treatments showed 145.3% and 47.99% high concentrations for H_2_O_2_, and 77.75% and 31.47% for MDA. At 90 DAT, no H_2_O_2_ and MDA were detected due to the complete death of plants. From these results, we assumed a higher toxic effect on hemp plants under those light treatments.

Previous studies explained the relationship between cell death and cannabinoid accumulation in hemp plants, especially the role of tetrahydrocannabinolic acid (THCA) (Shoyama et al., 2008; Islam et al., 2021b), cannabichromenic acid (CBCA) (Morimoto et al., 2007) as unique cell death mediators. H_2_O_2_ is well known as a signalling molecule and involved in many cases of plant cell death by increasing Ca^2+^ influx (Gechev and Hille, 2005). It was also stated that cannabinoids induce cell death independently of the H_2_O_2_-regulated cell death system, and this induction of cell death is not suppressed by pretreatment of H_2_O_2_-scavenging agents such as ascorbic acid (Morimoto et al., 2007). However, a positive correlation was also found among the cannabinoids, H_2_O_2_, and lipid peroxidation under several light spectra (Islam et al., 2021b).

Proline accumulation increased linearly as transplanting time increased up to 60 DAT, and then it tended to decrease for L1, L2 and L3 treatments, whereas L4 kept the uprising up to 90 DAT. The treatment L4 also showed a similar influencing strategy for TPC and TSC. In general, the synthesis and catabolism of proline help to buffer cellular redox potential and thus play a vital role in the stress adaptation of plants. It also scavenges free radicals and stabilizes sub-cellular structures despite playing the role of osmolyte under stress conditions (Hayat et al., 2012). Higher photosynthetic rate and stable pigments showed a compatible increment of proline up to 60 DAT in the present study. Similar results were also observed in case of ascorbic acid, TSC and sucrose. These results might be due to a higher accumulation of osmolytes which is described as an indicator of plant’s adaptive stress response (Islam et al., 2022). Carbohydrates play an active role in energy as well as principle criteria of cellular activity like cell division and growth in plants, where their concentration mostly depends on photosynthetic activity (Naithani et al., 2021). In the present experiment, a lower concentration of carbohydrates like TSC and sucrose under L3 and L4 treatments at 90 DAT indicates a stressful condition that might result from lower chlorophyll generation in plants (**Figure 3**). Moreover, L3 and L4 treatments showed higher photosynthetic activity at 60 DAT while tend to lower at 90 DAT. This contrariety may be due to the shortening life cycle of the plant as a result of the stress response, which is supported by the maximum concentration of proline and TPC at 90 DAT under L4 treatment.

The modulation of light quality significantly influenced the activities of antioxidant enzymes. In general, the increased ROS accumulation triggers the activities of antioxidant enzymes to prevent cell damage due to oxidative stress. The activity of CAT enzyme was subsequently higher in L3 and L4 treatments at 30 and 60 DAT (**Figure 6**). This higher activity was also partially supported by the ROS production under the treatments L3 and L4. In general, the CAT enzyme, a universal oxidoreductase, readily scavenges the excess ROS by the reduction of H_2_O_2_ to H_2_O and molecular oxygen (Manivannan et al., 2015). On the other hand, the increased activity of POD was also described as associated with the increased lipid peroxidation level (Shah et al., 2001). In the present study, the treatments L3 and L4 influenced both MDA and POD activities substantially. Similar results were described in a previous study where H_2_O_2_ and MDA were highly correlated to the higher activities of CAT and POD in the cannabis plants (Islam et al., 2021b). The higher ratio of red light under L3 and L4 treatments may have an influential role on higher enzymatic activities, as similar findings were reported in a previous study on wheat plants (Hui et al., 2017). Earlier studies also indicated that the regulation of ROS and their interaction with the antioxidant system are important mechanisms affecting plant growth and morphogenesis (Gupta and Agarwal, 2017; Xu et al., 2019).

During the initial stage of the cannabinoid pathway, a type III PKS enzyme named tetraketide synthase (TKS) activates with the help of olivetolic acid cyclase (OAC), a polyketide cyclase enzyme to form olivetolic acid (OA). OA reacts with geranyl pyrophosphate (GPP) with the help of CBGA synthase (CBGAS), a GPP: olivetolategeranyltransferase, to form CBGA. Later CBGA converts to THCA and CBDA, the biogenic acids of THC and CBD, by oxidocyclase enzymes (Fellermeier and Zenk, 1998; Sirikantaramas et al., 2007; Gagne et al., 2012; Magagnini et al., 2018). Despite little or no data available regarding the expression of these genes, in higher plants, some type III PKS such as chalone synthase (CHS), related to polyphenol accumulation, was substantially induced by light (Flores-Sanchez and Verpoorte, 2008). Plants use a complex photoreceptor system to perceive different wavelengths of light, activating various signal transduction cascades by transcriptional factors to regulate light responses. In this connection, it was also suggested that blue and UV-A light positively affected THC concentration in cannabis plants (Flores-Sanchez and Verpoorte, 2008). Our study also showed similar results as the treatment L2, containing UV-A, produced higher THC. Moreover, under L3 and L4 treatments, plants produced 83.24% and 83.74% less THC compared to L2. In the present study, L2 and L4 treatments produced the maximum amount of CBD, CBDA, and THCA, where the B:R proportion was higher than L3 treatment at the final growth stage (90 DAT). From the results, it was also plausible that the presence of UV-A and FR might have an influential role in cannabinoid accumulation. Besides, the lower accumulation of cannabinoid in L1 (except THC) might be due to the higher proportion of green light as it was assumed to influence cannabinoid negatively (Mahlberg and Hemphill, 1983). Environmental stress or a specific wavelength of light excites the photosensitizer that disturbs the balance between light harvesting and energy utilization, and this imbalance provokes ^3^Chl formation, which reacts with ground state triplet oxygen (^3^O_2_) to produce singlet oxygen (^1^O_2_). In addition, limited CO_2_ fixation due to stress conditions leads to a decrease in carbon reduction by the Calvin cycle and electron is transferred from photosystem I (PS I) to O_2_ to form $O_{2}^{•-}$ by the process called Mehler reaction, which is later converted to H_2_O_2_ (Karuppanapandian et al., 2011; Bőcskei-Antal et al., 2019). In the present experiment, plants manifested higher CBD (225%), CBDA (89.9%), and THCA (83.4%) in L4 treatment compared to L1 at 90 DAT. Besides, higher lipid peroxidation and enzyme activity in the treatment L4 were also observed at 30 and 60 DAT. However, the treatment L3 accumulated a bit lower cannabinoid despite having higher stress response activity such as H_2_O_2_, MDA, and enzymes. Cannabinoid are well-known secondary metabolites with high antioxidant activity (Mukhopadhyay et al., 2011; Raja et al., 2020). In the presence of higher ROS concentration, it might have a crucial role in bringing balance to the light-harvesting and energy utilization process. PCA analysis showed a positive correlation between H_2_O_2_ and cannabinoid at 30 and 60 DAT, clustered with the treatments L3 and L4 (**Figure 8**). Moreover, the plants died at 90 DAT under both L3 and L4 treatments. Although a previous study suggested that cannabinoid induces cell death independently of the H_2_O_2_-regulated cell death system (Morimoto et al., 2007), the present study partially supported the connection between ROS and cannabinoid accumulation.

## 5 Conclusions

LED light composition showed a stringent regulation of cannabis growth, development and metabolite accumulation. Among the various light treatments, L3 and L4 treatments at different growth period showed maximum shoot length and branch number, accumulation of Pro, AsA, TSC and sucrose, and higher activities of antioxidant enzymes like CAT and POD as compared to L1 and L2. An increasing trend of photosynthetic pigments and photosynthetic activity were also observed up to 60 DAT in L3 and L4 light compositions, which was further drastically reduced to a minimum level at 90 DAT. In addition, a higher tendency of H_2_O_2_ generation was recorded in L3 and L4 treatments, which influenced higher lipid peroxidation resulting in leaf necrosis and plant death in the later growth stage. These findings suggested that under L3 and L4 treatments, plants attained a relatively quick maturity due to higher ROS generation as evidenced by morpho-physiological data and PCA analysis, and playing a crucial role in cannabinoid like CBD, CBDA, and THCA accumulation. The results of this study can be used in cannabis industry to maximize the production of cannabinoid through the modulation of spectral composition.

## Data availability statement

The raw data supporting the conclusions of this article will be made available by the authors, without undue reservation.

## Author contributions

Conceptualization: MI and BR. Methodology: MI and BR. Formal analysis: MI and BR. Data curation: MI and BR. Statistical expertise: MI and BR. Writing−original draft preparation: MI and BR. Writing−review and editing: MR, EC, M-HW, J-DL, MH, and Y-SL. Visualization: MI and BR. Supervision: J-DL, MH, and Y-SL. Project administration: J-DL and Y-SL. Funding acquisition: J-DL and Y-SL. All authors have read and agreed to the published version of the manuscript.

## Acknowledgments

We gratefully acknowledge the Ministry of Science and ICT (MSIT, Korea, (support program: 2021-DD-UP-0379) and the BK21 FOUR program of the National Research Foundation (NRF, Korea) for providing support in the project. The authors also express their gratitude and profound appreciation to the CBF (Chuncheon Bioindustry Foundation, Korea) and Chuncheon City for their support in hemp variety breeding project.

## Conflict of interest statement

The authors declare that the research was conducted in the absence of any commercial or financial relationships that could be construed as a potential conflict of interest.

## Publisher’s note

All claims expressed in this article are solely those of the authors and do not necessarily represent those of their affiliated organizations, or those of the publisher, the editors and the reviewers. Any product that may be evaluated in this article, or claim that may be made by its manufacturer, is not guaranteed or endorsed by the publisher.
